# A minimising movement scheme for the *p*-elastic energy of curves

**DOI:** 10.1007/s00028-022-00791-w

**Published:** 2022-04-27

**Authors:** Simon Blatt, Christopher P. Hopper, Nicole Vorderobermeier

**Affiliations:** Departement of Mathematics, Paris Lodron Universität Salzburg, Hellbrunner Strasse 34, 5020 Salzburg, Austria

**Keywords:** Minimising movement, P-elastic energy for curves, Gradient flow, Approximate normal graphs, 53C44, 53A04

## Abstract

We prove short-time existence for the negative $L^2$-gradient flow of the *p*-elastic energy of curves via a minimising movement scheme. In order to account for the degeneracy caused by the energy’s invariance under curve reparametrisations, we write the evolving curves as approximate normal graphs over a fixed smooth curve. This enables us to establish short-time existence and give a lower bound on the solution’s lifetime that depends only on the $W^{2,p}$-Sobolev norm of the initial data.

## Introduction

For closed curves $\gamma :{\mathbb {R}}/{\mathbb {Z}} \rightarrow {\mathbb {R}}^n$ in the $W^{2,p}$-Sobolev class, we shall consider the energy

1.1 $$\begin{aligned} E (\gamma ) = \frac{1}{p} \int _{{\mathbb {R}} / {\mathbb {Z}}} |\kappa |^p \mathrm{d}s + \lambda \int _{{\mathbb {R}} / {\mathbb {Z}}} \mathrm{d}s, \end{aligned}$$

i.e. the sum of the *p*-elastic energy $E^{(p)}(\gamma ) = \tfrac{1}{p} \int _{{\mathbb {R}} / {\mathbb {Z}}} |\kappa |^p \mathrm{d}s$ and a positive multiple $\lambda >0$ of the length of the curve. A family of regular curves $\gamma =\gamma (t,x):[0,T) \times {\mathbb {R}} / {\mathbb {Z}} \rightarrow {\mathbb {R}}^n$ in the class

$$\begin{aligned} L^\infty \big ([0,T), W^{2,p}({\mathbb {R}} / {\mathbb {Z}}, {\mathbb {R}}^n)\big ) \cap W^{1,2} \big ([0,T),L^2 ({\mathbb {R}} / {\mathbb {Z}} , {\mathbb {R}}^n) \big ) \end{aligned}$$

is said to be a weak solution of the negative $L^2$-gradient flow of *E* if one has

1.2 $$\begin{aligned} \int _{0}^T \int _{{\mathbb {R}} / {\mathbb {Z}}} \langle \partial _t \gamma ,\psi \rangle \, \mathrm{d}s \mathrm{d}t = \int _{0}^T \delta _{\psi _t} E (\gamma _t) \, \mathrm{d}t \end{aligned}$$

for all test functions $\psi \in C^\infty _c ({\mathbb {R}} / {\mathbb {Z}} \times (0,T) , {\mathbb {R}}^n)$, i.e. the curve $\gamma$ satisfies $\partial _t \gamma = - \nabla _{L^2} E (\gamma )$ weakly, where $\delta _{\psi _t} E (\gamma _t) = \frac{d}{d\varepsilon } \left. E (\gamma _t + \varepsilon \psi _t) \right| _{\varepsilon = 0}$ is the first variation of the functional *E* at the curve $\gamma _t=\gamma (t, \,\cdot )$ in the direction of the test function $\psi _t=\psi (t, \,\cdot )$.

While the $L^2$-gradient flow of (1.1) has been extensively studied when $p=2$, both in the Euclidean (cf. [4, 5, 9, 15]) and manifold constrained (cf. [3, 15, 20]) settings, very little is know in the degenerate $p\ne 2$ case. For example, a second-order evolution equation has been considered for closed curves and planar networks (cf. [21, 22]) and the asymptotic of the flow has been studied away from degenerate points (cf. [24]); however, short-time existence for the Eq. (1.2) has yet to be established when $p\ne 2$. The aim of this article is to address both short and long time existence in the case $p>2$ for the geometric evolution (1.2) with initial data in the $W^{2,p}$-Sobolev class. Our approach is to rewrite the evolving curves as approximate normal graphs in order to utilise de Giorgi’s method of minimising movements (cf. [6]).

It is well-known that the invariance of the energy (1.1) under reparametrisations of the curve $\gamma$ leads to an evolution equation (1.2) that fails to be strongly parabolic (even in the $p=2$ case). This characteristic is in common with many other geometric evolution equations. For example, the failure of the strong ellipticity of the Ricci tensor is principally due to the second Bianchi identities.^1^ For this reason, short-time existence for the Ricci flow was originally established in [13] by appealing to the Nash–Moser implicit-function theorem (and the earlier exposition in [12]). DeTurck [7] subsequently showed that the Ricci flow is equivalent to an initial value problem for a parabolic system modulo the action of the diffeomorphism group of the underlying manifold. Thus, in a dramatic simplification that bypassed the Nash–Moser argument, one can pass from a weakly parabolic to a strongly parabolic system of equations by an appropriate choice of a 1-parameter family of diffeomorphisms. Perelman [23] also exploited the same diffeomorphism invariance in his gradient flow formalism for the Ricci flow. Versions of the DeTurck trick have also been used to obtain short-time existence for the mean curvature flow (cf. [2, 14]), the Willmore flow (cf. [16]) and the gradient flow of the elastic energy in both the Euclidean and manifold constrained cases.

In seeking to pass from the degenerate flow (1.1) to a strongly parabolic system, one can consider a time-dependent family of curves $\gamma _t = \gamma (t, \,\cdot \,)$ that are written as normal graphs over a given fixed smooth curve $\widetilde{\gamma }$, i.e. a family of curve of the form $\gamma _t = \widetilde{\gamma }+ \phi _t$ where $\phi _t = \phi (t, \,\cdot \,)$ is a perturbation normal to the fixed curve $\widetilde{\gamma }$. In this way, we obtain an evolution equation of the form

1.3 $$\begin{aligned} \int _{0}^T \int _{{\mathbb {R}} / {\mathbb {Z}}} \langle \gamma , \partial _t^\bot \psi \rangle \, \mathrm{d}s \mathrm{d}t = \int _{0}^T \delta _{\psi _t} E (\gamma _t) \, \mathrm{d}t \end{aligned}$$

for all test functions $\psi \in C^\infty _c ( (0,T) \times {\mathbb {R}} / {\mathbb {Z}} , {\mathbb {R}}^n)$, i.e. the curve $\gamma$ satisfies $\partial _t^\bot \gamma = - \nabla _{L^2} E (\gamma )$ weakly, where the normal velocity $\partial _t^\bot \gamma$ is the vector component of $\partial _t \gamma$ normal to the fixed curve $\widetilde{\gamma }$. Then in order to obtain a solution of (1.2) from a solution of (1.3), one can consider solutions $\Theta _t = \Theta (t, \,\cdot \,)$ of the ordinary differential equation

1.4 $$\begin{aligned} \left. \begin{aligned} \partial _t \Theta (t,x)&= F(t,\Theta (t,x)) \\ \Theta (0,x)&=x , \end{aligned} \right. \end{aligned}$$

where $F(t,y) = -\frac{\langle \partial _t \gamma (t,y), \gamma '(t,y)\rangle }{|\gamma '(t,y)|^2}$ and $\gamma$ is a solution of (1.3). The existence of ODE solutions can thus be established on a time interval $0 \le t < \varepsilon$ for some $\varepsilon >0$ independent of the initial point $x\in {\mathbb {R}}/{\mathbb {Z}}$. Therefore, if $\Theta _t = \Theta (t, \,\cdot \,)$ is a solution of (1.4) and $\gamma _t = \gamma (t,\,\cdot \,)$ is a solution of (1.3), the composition $\gamma _t \circ \Theta _t$ is a solution of (1.2). By taking this approach, one can thus establish the existence of solutions for geometric flows with initial data in the $C^{2,\alpha }$-Hölder class even though the original equations may be ill-defined (see, e.g. [10, 19, 26]). In fact, a recent paper by LeCrone, Shao and Simonett [18] showed how to reduce the regularity of the initial data to the $C^{1,\alpha }$-Hölder class.

In order to carry out the aforementioned programme, one has to guarantee that a given initial curve $\Gamma$ can be written as a normal graph over a fixed smooth curve $\widetilde{\gamma }$. Since it is not possible to write every curve $\Gamma$ in the $W^{2,p}$-Sobolev class as a normal graph over a smooth curve, we are spurred on to introduce the notion of a unit quasi-tangent $\tau$ (cf. Definition 2.4) which then defines an *approximate tangential projection* $P_\tau ^T$ and an *approximate normal projection* $P_\tau ^\bot = I - P_\tau ^T$ (cf. Definition 2.6). In which case one can write the curve $\Gamma$ as equal to $\widetilde{\gamma }+ \Phi$ up to a reparametrisation, i.e. as an approximate normal graph over a smooth curve $\widetilde{\gamma }$ with some perturbation $\Phi$ orthogonal to $\tau$ (cf. Lemma 2.12). Then by applying a minimising movements scheme, it is possible to establish the existence of a family of curves of the form $\gamma _t = \widetilde{\gamma }+ \phi _t$, for a suitable perturbation $\phi _t$ orthogonal to $\tau$, that satisfies $\partial _t^\bot \gamma = - \nabla _{L^2} E(\gamma )$ weakly. Indeed, we have:

### Theorem 1.1

(Existence) For any given initial curve $\Gamma \in W^{2,p}({\mathbb {R}} /L {\mathbb {Z}}, {\mathbb {R}}^n)$ parametrised by arc-length there exists a smooth curve $\widetilde{\gamma }\in C^\infty ({\mathbb {R}}/L{\mathbb {Z}},{\mathbb {R}}^n)$ parametrised by arc-length, a quasi-tangent $\tau$ to the curve $\widetilde{\gamma }$, a finite time $T=T(p,\lambda , E(\Gamma ))>0$ and a family of perturbations $\phi$ in the class

$$\begin{aligned} L^\infty \big ([0,T),W^{2,p}({\mathbb {R}}/L{\mathbb {Z}},{\mathbb {R}}^n) \big ) \cap \big (W^{1,2} \cap C^{1/ 2}\big ) \big ([0,T),L^2({\mathbb {R}}/L{\mathbb {Z}},{\mathbb {R}}^n) \big ) \end{aligned}$$

which are orthogonal to $\tau$ such that the family of curves

$$\begin{aligned} \gamma (t,s) = \widetilde{\gamma }(s) + \phi (t,s),\quad 0\le t<T, \end{aligned}$$

satisfies the initial condition $\gamma (0, \,\cdot \, ) = \Gamma \circ \sigma$ for some reparametrisation $\sigma$ of ${\mathbb {R}}/L{\mathbb {Z}}$ and

1.5 $$\begin{aligned} \int _{0}^T \int _{{\mathbb {R}} / L{\mathbb {Z}}} \langle \partial _t^\bot \gamma ,\psi \rangle \, \mathrm{d}s \mathrm{d}t = - \int _{0}^T \delta _{\psi _t} E (\gamma _t) \mathrm{d}t \end{aligned}$$

for all test functions $\psi \in C^\infty _c((0,T) \times {\mathbb {R}} / L{\mathbb {Z}} ,{\mathbb {R}}^n )$ orthogonal to $\tau$.

Note that the time of existence only depends on the energy of the initial curve. So we are very close to restarting the flow and deduce long time existence. We discuss in the final section, why this is not as straightforward as it might seem.

By assuming the solution has some additional regularity, one can show that Eq. (1.5) holds for all test functions (i.e. our solution solves the original weak form of the desired evolution equation).

### Corollary 1.2

If the solution $\gamma (t, \,\cdot \, )$ of Theorem 1.1 belongs to the $W^{3,p}$-Sobolev class for almost all $0\le t<T$, then

$$\begin{aligned} \int _0^T \int _{{\mathbb {R}} / L{\mathbb {Z}}} \langle \partial ^\bot _t \gamma ,\psi \rangle \mathrm{d}s \mathrm{d}t = - \int _0^T \delta _{\psi _t} E(\gamma ) \mathrm{d}t \end{aligned}$$

for all test functions $\psi \in C^\infty _c ((0,T) \times {\mathbb {R}}/L {\mathbb {Z}}, {\mathbb {R}}^n)$.

## Minimising movements scheme

It is remarked by De Giorgi [6] that a generalised minimising movements scheme could provide a formalism for the existence of steepest descent curves of a functional in a metric space. In order to establish the existence of weak solutions for (1.2), we need to take care of the twofold degeneracies arising from the invariance of (1.1) under curve reparametrisation and the fact that $p>2$. We tackle this issue by writing the evolving curve as an approximate normal graph over a fixed smooth curve so that we can work with the normal velocity (rather than the time derivative) of the evolving curve.

### Tubular neighbourhoods

For an embedded $C^k$-submanifold ${\mathcal {M}}$ of ${\mathbb {R}}^n$ without boundary, the normal bundle $(T{\mathcal {M}})^\perp \rightarrow {\mathcal {M}}$ is only of the class $C^{k-1}$. If we define the ‘endpoint’ map $E:(T{\mathcal {M}})^\perp \rightarrow {\mathbb {R}}^n$ by sending

$$\begin{aligned} (x,v) \mapsto x+v \end{aligned}$$

and assume $k\ge 2$, one can use the inverse function theorem to show that there exists a tubular neighbourhood *U* of ${\mathcal {M}}$ in ${\mathbb {R}}^n$ that is the diffeomorphic image under the $C^{k-1}$-map *E* of an open neighbourhood of the zero section of $(T{\mathcal {M}})^\perp$. Moreover, the squared distance function $\zeta (x) = \frac{1}{2} \text {dist}(x,{\mathcal {M}})^2$ is a function in $C^{k}(U)$ (cf. [11]) and the Hessian matrix $\nabla ^2 \zeta (x)$ represents the orthogonal projection on the normal space to ${\mathcal {M}}$ at a point *x* (cf. [1, p. 704]). Of course, these results no longer hold in the case $k=1$, i.e. when the inverse function theorem is not applicable.

### Approximate normal graphs

As the normal bundle of an embedded $W^{2,p}$-curve in ${\mathbb {R}}^n$ is only of the class $W^{1,p}$, one cannot directly apply the standard methods of Sect. 2.1. In particular, we need to overcome the loss of regularity on the level of the tangent space in order to write the solution of our equation locally as a graph over a fixed smooth curve. This problem can be resolved by regularising the tangent using Friedrichs mollifiers (whilst taking into consideration the size of the constructed tubular neighbourhood). We will call this smoothened tangent *quasi-tangent*.

#### Definition 2.1

A function $\eta \in C^\infty ({\mathbb {R}})$ is called a *mollifier* if it satisfies the conditions: (i) $\eta \ge 0$ on ${\mathbb {R}}$, (ii) $\eta (x) = 0$ for all $|x| \ge 1$, and (iii) $\int _{\mathbb {R}}\eta (x) \mathrm{d}x = 1$. The associated *rescaled mollifier* is the function $\eta _\varepsilon (x) = \frac{1}{\varepsilon }\eta (\frac{x}{\varepsilon })$ for any $\varepsilon >0$.

Now consider a curve $\gamma \in W^{2,p}({\mathbb {R}} / {\mathbb {Z}}, {\mathbb {R}}^n)$ parametrised by arc-length. The *mollification* of $\gamma$ is defined to be the function

$$\begin{aligned} \gamma _\varepsilon (x) = (\gamma *\eta _\varepsilon )(x) = \int _{\mathbb {R}}\gamma (x-y) \eta _\varepsilon (y) \mathrm{d}y , \end{aligned}$$

i.e. the convolution of the given curve $\gamma$ and the rescaled mollifier $\eta _\varepsilon$.

For the mollified curve $\gamma _\varepsilon$, we derive the following well-known estimates. Firstly, from the mean value theorem and the Sobolev embeddings, we find that

2.1 $$\begin{aligned} | \gamma _\varepsilon (x) - \gamma (x) |&= \left| \int _{{\mathbb {R}}} (\gamma (x-y) - \gamma (x) ) \eta _\varepsilon (y) \mathrm{d}y\right| \nonumber \\&\le \varepsilon \Vert \gamma '\Vert _{L^\infty } \nonumber \\&\le C \varepsilon \Vert \gamma '\Vert _{W^{1,p}} . \end{aligned}$$

Likewise, we find that

2.2 $$\begin{aligned} | \gamma '_\varepsilon (x) - \gamma '(x) |&= \left| \int _{{\mathbb {R}}} \eta _\varepsilon (y) (\gamma '(x-y) - \gamma '(x) ) \mathrm{d}y\right| \nonumber \\&\le \sqrt{\varepsilon } \Vert \gamma '\Vert _{C^{1/2}} \nonumber \\&\le C \sqrt{\varepsilon } \Vert \gamma '\Vert _{W^{1,p}}. \end{aligned}$$

For higher derivatives, we can use the Sobolev embeddings, Hölder’s inequality and integration by parts to obtain the $L^\infty$-bound

2.3 $$\begin{aligned} | \gamma ^{(k+2)}_\varepsilon (x) |&=\left| \int _{{\mathbb {R}}} \eta _{\varepsilon }^{(k)}(y) \gamma ''(x-y) \mathrm{d}y\right| \nonumber \\&\le \Vert \eta ^{(k)}_{\varepsilon } \Vert _{L^q} \Vert \gamma ''\Vert _{L^p} \nonumber \\&\le C \varepsilon ^{-k-1+\frac{1}{q}} \Vert \gamma ''\Vert _{L^p} \nonumber \\&= C \varepsilon ^{-k - \frac{1}{p}} \Vert \gamma ''\Vert _{L^p} \end{aligned}$$

for integers $k\ge 0$ with $\frac{1}{p} + \frac{1}{q} = 1$.

We will use the next lemma to fix the smoothing parameter $\varepsilon .$

#### Lemma 2.2

If for an $M> 0$, we have a curve $\gamma \in W^{2,p}({\mathbb {R}} / {\mathbb {Z}}, {\mathbb {R}}^n)$ parametrised by arc-length which satisfies $\Vert \gamma '\Vert _{W^{1,p}} \le M$, then there exists an $\varepsilon =\varepsilon (p,M) >0$ such that the unit tangent $\tau = \frac{ \gamma '_{\varepsilon }}{|\gamma '_{\varepsilon }|}$ satisfies

2.4 $$\begin{aligned} \Vert \tau - \gamma ' \Vert _{L^\infty } \le \frac{1}{4} .\end{aligned}$$

#### Proof

Using (2.2), we get $\Vert \gamma _{\varepsilon }' - \gamma ' \Vert _{L^\infty } \le C \sqrt{\varepsilon }\Vert \gamma '\Vert _{W^{1,p}}.$ As the retraction map $\Pi :x \mapsto \frac{x}{|x|}$ is locally Lipschitz on ${\mathbb {R}}^n\backslash \{0\}$, the tangent $\tau = \Pi (\gamma '_{\varepsilon }) = \frac{ \gamma '_{\varepsilon }}{|\gamma '_{\varepsilon }|}$ to the mollified curve $\gamma _{\varepsilon }$ satisfies (2.4) for some $\varepsilon >0$ sufficiently small. $\square$

#### Corollary 2.3

If for an $M> 0$, we have a curve $\gamma \in W^{2,p}({\mathbb {R}} / {\mathbb {Z}}, {\mathbb {R}}^n)$ parametrised by arc-length which satisfies $\Vert \gamma '\Vert _{W^{1,p}} \le M$, then for an $\varepsilon =\varepsilon (p,M)>0$ as in Lemma 2.2 the mollified curve $\gamma _\varepsilon$ has a unit tangent map $\tau :{\mathbb {R}} / {\mathbb {Z}} \rightarrow S^{n-1}$ that is smooth and satisfies

2.5 $$\begin{aligned} \Vert \tau '\Vert _{L^\infty } , \Vert \tau ''\Vert _{L^\infty } \le C \end{aligned}$$

for a constant $$C=C(p,M)>0$$.

#### Definition 2.4

We say $\tau$ is *unit quasi-tangent* to the $W^{2,p}$-curve $\gamma$ if it is the unit tangent to the mollified curve $\gamma _\varepsilon$ for some $\varepsilon =\varepsilon (p,M)>0$ as in Lemma 2.2.

#### Definition 2.5

We denote by $P^T _v w = \langle w, \frac{v}{|v|} \rangle \frac{v}{|v|}$ the orthogonal projection of *w* onto the line ${\mathbb {R}} v$ for any vectors $v,w \in {\mathbb {R}}^n$. Likewise, we denote by $P^\bot _v w = w - P^T _v w$ the orthogonal projection of *w* onto the orthogonal complement $({\mathbb {R}} v)^\bot$ of the line ${\mathbb {R}} v$.

#### Definition 2.6

If $\tau$ is unit quasi-tangent to a $W^{2,p}$-curve $\gamma$, we denote by $(W^{2,p})^T_{\tau }$ (resp. $(W^{2,p})^\bot _{\tau }$) the set of all $w \in W^{2,p}({\mathbb {R}} / {\mathbb {Z}}, {\mathbb {R}}^n)$ such that $P_\tau ^\bot w =0$ a.e. (resp. $P_\tau ^T w= 0$ a.e.).

We will now prove the following statement that gives a lower bound on the thickness of the set of regular curves around $\gamma$.

#### Lemma 2.7

If for an $M> 0$ we have a curve $\gamma \in W^{2,p}({\mathbb {R}} / {\mathbb {Z}}, {\mathbb {R}}^n)$ parametrised by arc-length which satisfies $\Vert \gamma '\Vert _{W^{1,p}} \le M$, then there exists a constant $K=K(p,M)>0$ and a unit quasi-tangent $\tau$ to the curve $\gamma$ such that the curve $\gamma + \phi$ satisfies

$$\begin{aligned} \inf _{x\in {\mathbb {R}} / {\mathbb {Z}}}\langle \gamma ' + \phi ', \tau \rangle \ge \frac{1}{2} \end{aligned}$$

and hence

$$\begin{aligned} \inf _{x\in {\mathbb {R}} / {\mathbb {Z}}} |\gamma '(x) + \phi '(x)| \ge \frac{1}{2} \end{aligned}$$

for each $\phi \in (W^{2,p})^\bot _{\tau }$ with $\Vert \phi \Vert _{L^\infty } \le K$. In particular, $\gamma +\phi$ is a regular curve.

#### Proof

We first note that $\langle \gamma ', \tau \rangle = |\gamma '|^2 + \langle \gamma ', \tau - \gamma '\rangle \ge 1 - |\tau - \gamma '| \ge \frac{3}{4}$ by Lemma 2.2 and the fact that $|\gamma '|=1$. Upon differentiating the orthogonality condition $\langle \phi , \tau \rangle = 0$, we get $\langle \phi ', \tau \rangle = - \langle \phi , \tau ' \rangle$. In which case the estimate (2.5) implies that $|\langle \phi ', \tau \rangle | = |\langle \phi , \tau '\rangle | \le C \Vert \phi \Vert _{L^\infty } \le \frac{1}{4}$ whenever $\Vert \phi \Vert _{L^\infty } \le \frac{1}{4 C } = K$. Thus,

$$\begin{aligned} \langle \gamma ' + \phi ', \tau \rangle \ge \frac{3}{4} - \frac{1}{4} = \frac{1}{2} \end{aligned}$$

whenever $\Vert \phi \Vert _{L^\infty } \le K$, i.e. $\gamma + \phi$ is a regular curve. As $\tau$ is of unit length, we also have $|\gamma ' + \phi '| \ge \frac{1}{2}$ on ${\mathbb {R}} / {\mathbb {Z}}$. $\square$

We will now deduce the following lower bound for the $L^p$-norm of the curvature of a curve $\widetilde{\gamma }+\phi$ in terms of the $L^p$-norm of the second derivative of $\phi$. This bound extends to our situation the well-known analogous result for the case of a real normal graph over a smooth curve.

#### Lemma 2.8

If for an $M> 0$ we have a curve $\gamma \in W^{2,p}({\mathbb {R}} / {\mathbb {Z}}, {\mathbb {R}}^n)$ parametrised by arc-length which satisfies $\Vert \gamma '\Vert _{W^{1,p}} \le M$ and a unit quasi-tangent $\tau$ to the curve $\gamma$, then for the constant $K=K(p,M)>0$ from Lemma 2.7 we have for each $\phi \in (W^{2,p})^\bot _{\tau }$ with $\Vert \phi \Vert _{L^\infty } \le K$

$$\begin{aligned} | v| \le C |P_{ \gamma '+ \phi '}^{\bot } v| \end{aligned}$$

for all $v\in {\mathbb {R}}^n$ pointing in an approximate normal direction and

$$\begin{aligned} \int _{{\mathbb {R}}/{\mathbb {Z}}} | \phi ''|^p \mathrm{d}s \le C \bigg ( 1+ \int _{{\mathbb {R}}/{\mathbb {Z}}} |\kappa _{ \gamma + \phi }|^p \mathrm{d}s \bigg ) \end{aligned}$$

for some $$C=C(M,p)$$.

#### Proof

Since $\langle \gamma ' + \phi ', \tau \rangle \ge \frac{1}{2}$ from Lemma 2.7 and $| \gamma ' + \phi '| \le | \gamma '|+|\phi '| \le 1+\Lambda$, we see that $\langle \frac{ \gamma ' + \phi '}{| \gamma ' + \phi '|} , \tau \rangle \ge \frac{1}{2} \frac{1}{1+\Lambda } .$ Hence, the angle between $\gamma ' + \phi '$ and $\tau$ is bounded strictly away from $\frac{\pi }{2}$. In which case we have

$$\begin{aligned} |v| \le C |P_{ \gamma '+ \phi '}^{\bot } v| \end{aligned}$$

for all $v \in {\mathbb {R}}^n$ pointing in an approximate normal direction.

For the second estimate, we recall the curvature formula given by

$$\begin{aligned} \kappa _{ \gamma + \phi } = \frac{P^\bot _{ \gamma ' + \phi '} ( \gamma '' + \phi '')}{| \gamma ' + \phi '|^2} .\end{aligned}$$

Now by the triangle inequality, we see that

$$\begin{aligned} \left| P^\bot _{ \gamma ' + \phi '} ( \phi '' ) \right| \le \left| P^\bot _{ \gamma ' + \phi '} ( \gamma ''+ \phi '' ) \right| + \left| P^\bot _{ \gamma ' + \phi '} ( \gamma '' ) \right| \le C (|\kappa _{ \gamma + \phi }| + |\gamma ''|) , \end{aligned}$$

since $| \gamma ' + \phi '|\le | \gamma '| + |\phi '|\le 1 +\Lambda$. To control the tangential part $P^T_\tau \phi ''$, we differentiate the equation $\langle \phi , \tau \rangle =0$ twice to get $\langle \phi '' , \tau \rangle = - 2 \langle \phi ', \tau ' \rangle - \langle \phi , \tau '' \rangle .$ It then follows that

$$\begin{aligned} |P^T_{\tau } \phi ''| = | \langle \phi '' , \tau \rangle | \le | \langle \phi , \tau '' \rangle | + 2 |\langle \phi ', \tau ' \rangle | \le C (K+ \Lambda ) , \end{aligned}$$

since both $\tau '$ and $\tau ''$ are bounded by Corollary 2.3. In combining both the tangential and normal parts of $\phi ''$ and using the fact that the angle between $\gamma ' + \phi '$ and $\tau$ is bounded strictly away from $\tfrac{\pi }{2}$, we find that

$$\begin{aligned} |\phi ''| \le C( |P^\bot _{\gamma ' + \phi '} \phi ''| + |P^T_{\tau } \phi ''| ) \le C (1+ | \gamma '' | + |\kappa _{ \gamma + \phi }|) \end{aligned}$$

from which the desired integral estimate follows (since $\Vert \gamma ''\Vert _{L^p} \le M$ by Lemma 2.12). $\square$

Next we show that there exists a good substitute for the nearest neighbourhood projection which yields a local tubular neighbourhood. We also obtain a lower bound on thickness of the tubular neighbourhood that only depends on the $W^{2,p}$-norm of the curve.

#### Definition 2.9

If $\tau$ is a unit quasi-tangent to a $W^{2,p}$-curve $\gamma$, the $(n-1)$-dimensional subspace

$$\begin{aligned} {\mathcal {N}}_{x_0}=\{v \in {\mathbb {R}}^n: P^T_{\tau (x_0)} v =0\} \end{aligned}$$

is called an *approximate normal space* to $\gamma$ at a given fixed point $x_0 \in {\mathbb {R}} / {\mathbb {Z}}$.

By considering the map $H_{x_0} :B_{\delta }(x_0) \times {\mathcal {N}}_{x_0} \rightarrow {\mathbb {R}}^n$ given by

2.6 $$\begin{aligned} (x,v) \mapsto \gamma (x) + P^\bot _{\tau (x)} v \end{aligned}$$

for some $0<\delta <1$, we obtain the following:

#### Lemma 2.10

If for an $M> 0$, we have a curve $\gamma \in W^{2,p}({\mathbb {R}} / {\mathbb {Z}}, {\mathbb {R}}^n)$ parametrised by arc-length which satisfies $\Vert \gamma '\Vert _{W^{1,p}} \le M$ and a unit quasi-tangent $\tau$ to the curve $\gamma$, then there exists a sufficiently small constant $\delta = \delta (p,M) >0$ such that (2.6) maps $B_\delta (x_0) \times B_\delta (0)$ diffeomorphically onto its image and

2.7 $$\begin{aligned} B_{ \delta / 4} \big (\gamma (B_{ \delta / 4}(x_0))\big ) \subset H_{x_0} \big (B_\delta (x_0) \times B_\delta (0)\big ) .\end{aligned}$$

#### Proof

We first show that $H_{x_0}$ is a local diffeomorphism by way of the inverse function theorem. To do so, we calculate the partial derivatives

$$\begin{aligned} \frac{\partial H_{x_0}}{\partial x}&= \gamma '(x) - \langle v, \tau '(x) \rangle \tau (x) - \langle v, \tau (x) \rangle \tau '(x) \\ \frac{\partial H_{x_0}}{\partial v}&= P^\bot _{\tau (x)} v = v + P^\bot _{\tau (x)} v - P^\bot _{\tau (x_0)} v ,\end{aligned}$$

since $v \in {\mathcal {N}}_{x_0}$ and hence $P^\bot _{\tau (x_0)} v=v$. Then from the estimates (2.4) and (2.5) together with the Sobolev embedding $W^{2,p}(B_\delta (x_0),{\mathbb {R}}^n) \hookrightarrow C^{1,1-\frac{1}{p}} (B_\delta (x_0),{\mathbb {R}}^n)$, we find that

$$\begin{aligned} \Big | \frac{\partial H_{x_0}}{\partial x} - \tau (x_0) \Big |&\le | \gamma '(x_0) - \tau (x_0)| + C|v| +|\gamma '(x) - \gamma '(x_0)| \\&\le \frac{1}{4} + C |v| + C \delta ^{1-\frac{1}{p}} \end{aligned}$$

for some constant $C=C(p,M)>0$. By taking some $\delta > 0$ sufficiently small (depending only on *p* and *M*), we have

$$\begin{aligned} \Big | \frac{\partial H_{x_0}}{\partial x} - \tau (x_0) \Big | \le \frac{1}{2} \end{aligned}$$

for all $x\in B_\delta (x_0) \subset {\mathbb {R}}/{\mathbb {Z}}$ and $v \in B_\delta (0)\subset {\mathcal {N}}_{x_0}$. Likewise, whenever $\delta > 0$ is sufficiently small, we also have

$$\begin{aligned} \Big | \frac{\partial H_{x_0}}{\partial v} - v \Big | \le \frac{1}{2} \end{aligned}$$

for all $$(x,v) \in B_\delta (x_0) \times B_\delta (0)$$.

Let us now assume that $$\tau (x_0) = e_1$$ without loss of generality. From the above estimates, we see that the Jacobi matrix $$ D H_{x_0}$$ satisfies

2.8 $$\begin{aligned} \Vert D H_{x_0} - I\Vert \le \frac{1}{2} , \end{aligned}$$

where $\Vert \cdot \Vert$ denotes the operator norm. Therefore, $DH_{x_0}$ is invertible and so $H_{x_0}$ maps $B_{\delta }(x_0) \times B_{\delta }(0)$ diffeomorphically onto its image by the inverse function theorem. Moreover, (2.8) implies that

$$\begin{aligned} |H_{x_0}(z_1)- H_{x_0}(z_2)|&= \left| \int _{0}^1 D H_{x_0} (z_2 + \theta (z_1 - z_2)) (z_1-z_2) \mathrm{d}\theta \right| \\&\ge |z_1 - z_2 | - \tfrac{1}{2} |z_1 - z_2| \\&= \tfrac{1}{2} |z_1 - z_2| . \end{aligned}$$

In which case the map $H_{x_0}$ is bi-Lipschitz and hence injective on $B_{\delta }(x_0) \times B_{\delta }(0)$. From the fact that ${{\,\mathrm{dist}\,}}( \partial (B_{\delta } (x_0) \times B_{\delta } (0)), B_{ \delta / 2} (x_0) \times \{0\} ) \ge \frac{\delta }{2}$ and the latter bi-Lipschitz estimate, we have

$$\begin{aligned} {{\,\mathrm{dist}\,}}\Big ( H_{x_0}\big (\partial (B_{\delta } (x_0) \times B_{\delta } (0))\big ), \gamma (B_{ \delta /2} (x_0))\Big ) \ge \frac{\delta }{4} \end{aligned}$$

which then gives (2.7). $\square$

We can now use Lemma 2.10 to show that any $W^{2,p}$-curve $\gamma$ can be written as an approximate normal graph over a given $W^{2,p}$-curve $\widetilde{\gamma }$ whenever the curves are $C^1$-close to each other.

#### Lemma 2.11

If for an $M> 0$, we have a curve $\gamma \in W^{2,p}({\mathbb {R}} / {\mathbb {Z}}, {\mathbb {R}}^n)$ parametrised by arc-length that satisfies $\Vert \gamma '\Vert _{W^{1,p}} \le M$ and a unit quasi-tangent $\tau$ to the curve $\gamma$, then there exists a sufficiently small constant $\rho =\rho (p,M) >0$ such that for each curve $\widetilde{\gamma }\in W^{2,p}({\mathbb {R}} / {\mathbb {Z}}, {\mathbb {R}}^n)$ satisfying $\Vert \gamma - \widetilde{\gamma }\Vert _{C^1} \le \rho$ we have some $\phi \in (W^{2,p})^\bot _\tau$ and a reparametrisation $\sigma$ of ${\mathbb {R}}/ {\mathbb {Z}}$ for which $\widetilde{\gamma }\circ \sigma = \gamma + \phi .$

#### Proof

Firstly, choose $\delta >0$ as in Lemma 2.10. Since ${\mathbb {R}} / {\mathbb {Z}}$ is compact, there exist points $x_1$, ..., $x_\ell$ in ${\mathbb {R}} / {\mathbb {Z}}$ such that the balls $B_{ \delta / 4 } (x_1)$, ..., $B_{ \delta / 4 } (x_\ell )$ cover ${\mathbb {R}} / {\mathbb {Z}}$. Let the mappings $H_{x_j}$ for $j=1,\ldots ,\ell$ be defined by (2.6) and let $\Pi _{x_j}:B_{ \delta / 4} \big ( \gamma (B_{ \delta / 4} (x_j ) ) \big ) \rightarrow {\mathbb {R}} /{\mathbb {Z}}$ be the corresponding retraction maps given by

$$\begin{aligned} \Pi _{x_j} = \pi \circ H_{x_j}^{-1} \end{aligned}$$

where $\pi :B_{\delta } (x_j) \times {\mathcal {N}}_{x_j} \rightarrow {\mathbb {R}} / {\mathbb {Z}}$ sends $(x,v) \mapsto x$, i.e. the projection onto the first coordinate. We can then set

2.9 $$\begin{aligned} \sigma (x) = \Pi _{x_j} ( \widetilde{\gamma }(x)) \end{aligned}$$

for any $x \in B_{\delta } (x_j)$ in order to get a well-defined $C^1$-mapping. Note that the affine subspaces $\gamma (x) + {\mathcal {N}}_x$ and not their parametrisations determine the projections $H_{x_j}$. Hence, they agree for different $x_j$ if the domains of definition overlap. Furthermore, from the inverse function theorem applied to $\Pi _{x_j}$ and the estimate (2.8) we see that $\sigma '(x) >0$ whenever $\rho >0$ is sufficiently small (i.e. $\sigma$ is bi-Lipschitz). In addition, by setting $\widetilde{\phi }= \widetilde{\gamma }- \gamma \circ \sigma$ we see from (2.6) that $\widetilde{\phi }$ belongs to $(W^{2,p})^\bot _{\tau \circ \sigma }$. Therefore, $\gamma \circ \sigma$ is a regular curve equal to $\widetilde{\gamma }- \widetilde{\phi }$. In order to change the roles of $\gamma$ and $\widetilde{\gamma }$, we apply the inverse function theorem to $\sigma$ to justify the reparametrisation $\widetilde{\gamma }\circ \sigma ^{-1} = \gamma \circ \sigma \circ \sigma ^{-1} + \widetilde{\phi }\circ \sigma ^{-1} = \gamma + \phi$, where we set $\phi = \widetilde{\phi }\circ \sigma ^{-1} \in (W^{2,p})^\bot _\tau$.

$$\square $$

Using the above lemma, we can write every $W^{2,p}$-curve $\gamma$ as an approximate normal graph over a smooth curve $\widetilde{\gamma }$. Be aware that from now on till the end of this article we consider normal graphs over the curve $\tilde{\gamma }$ instead of $\gamma .$

#### Lemma 2.12

Let $\gamma \in W^{2,p}({\mathbb {R}} / {\mathbb {Z}}, {\mathbb {R}}^n)$ be a curve parametrised by arc-length. For every $\varepsilon _0>0$, there exists a smooth curve $\widetilde{\gamma }\in C^\infty ({\mathbb {R}} / {\mathbb {Z}}, {\mathbb {R}}^n)$ parametrised by arc-length with $\Vert \gamma - \widetilde{\gamma }\Vert _{W^{2,p}} \le \varepsilon _0$, a unit quasi-tangent $\tau$ to the curve $\tilde{\gamma }$ and some $\phi \in (W^{2,p})^\bot _\tau$ such that

2.10 $$\begin{aligned} \gamma \circ \sigma = \widetilde{\gamma }+ \phi \end{aligned}$$

for a reparametrisation $\sigma$ of ${\mathbb {R}}/ {\mathbb {Z}}$.

#### Proof

Firstly, there exists a smooth curve $\widetilde{\gamma }\in C^\infty ({\mathbb {R}}/{\mathbb {Z}},{\mathbb {R}}^n)$ parametrised by arc-length such that

$$\begin{aligned} \Vert \tilde{\gamma }- \gamma \Vert _{W^{2,p}} \le \varepsilon _0 \end{aligned}$$

by the density of $C^\infty ({\mathbb {R}}/{\mathbb {Z}},{\mathbb {R}}^n)$ in $W^{2,p}({\mathbb {R}}/{\mathbb {Z}},{\mathbb {R}}^n)$. Moreover, we have

$$\begin{aligned} \Vert \gamma -\widetilde{\gamma }\Vert _{C^1} \le C \varepsilon _0 = \rho \end{aligned}$$

by the Sobolev embeddings. Thus, by taking some $\varepsilon _0>0$ sufficiently small, Lemma 2.11 implies that there exists some $\phi \in (W^{2,p})^\bot _\tau$ and a reparametrisation $\sigma$ of ${\mathbb {R}}/ {\mathbb {Z}}$ such that $\gamma \circ \sigma = \widetilde{\gamma }+ \phi$. $\square$

The representation of $\gamma$ by a normal graph $\phi$ over $\widetilde{\gamma }$ we obtain from Lemma 2.12 satisfies the following $C^1$ estimates. These enable us to control the second derivative of $\phi$ by the curvature of $\gamma$ using Lemma 2.8.

#### Corollary 2.13

For the decomposition (2.10), there exists a constant $C>0$ depending on an upper bound *M* on $\Vert \gamma '\Vert _{W^{1,p}}$ and *p* such that

$$\begin{aligned} \Vert \phi \Vert _{L^\infty } \le C \Vert \gamma - \widetilde{\gamma }\Vert _{L^\infty } \quad \text {and} \quad \Vert \phi '\Vert _{L^\infty } \le C (1+ \Vert \gamma ' - \widetilde{\gamma }' \Vert _{L^\infty } ) .\end{aligned}$$

#### Proof

From the construction of $\sigma$ given by (2.9), we see that

$$\begin{aligned} |\sigma (x) - x| = |\sigma (x) - \sigma \circ \sigma ^{-1} (x)| \le \Vert \sigma '\Vert _{L^\infty } |x-\sigma ^{-1}(x)| \end{aligned}$$

and

$$\begin{aligned} |x-\sigma ^{-1}(x)| = |\Pi _{x_j}(\widetilde{\gamma }(x)) - \Pi _{x_j}( \gamma (x)) | \le \big ( \max _j \Vert D \Pi _{x_j}\Vert _{L^\infty } \big ) |\widetilde{\gamma }(x) - \gamma (x) | ,\end{aligned}$$

since there exists some ball $B_{\delta }(x_j)$ such that $x = \Pi _{x_j}( \widetilde{\gamma }(x))$. As we have

$$\begin{aligned} |\phi (x)| = | \gamma (\sigma (x)) - \widetilde{\gamma }(x) | \le | \gamma (\sigma (x)) - \gamma (x) | + | \gamma (x) - \widetilde{\gamma }(x) | \end{aligned}$$

and $| \gamma (\sigma (x)) - \gamma (x) | \le \Vert \gamma '\Vert _{L^\infty } |\sigma (x) - x|$, it follows that

$$\begin{aligned} \Vert \phi \Vert _{L^\infty } \le (1+ C \Vert \gamma '\Vert _{W^{1,p}} )\Vert \gamma - \widetilde{\gamma }\Vert _{L^\infty } \end{aligned}$$

by the Sobolev embeddings. In addition, we have

$$\begin{aligned} \Vert \phi '\Vert _{L^\infty } = \Vert (\gamma \circ \sigma ) ' - \widetilde{\gamma }' \Vert _{L^\infty }&\le \Vert \gamma ' - \widetilde{\gamma }' \Vert _{L^\infty } + \Vert \gamma ' \Vert _{L^\infty } + \Vert (\gamma \circ \sigma )'\Vert _{L^\infty } \\&\le \Vert \gamma ' -\widetilde{\gamma }'\Vert _{L^\infty } + C \Vert \gamma '\Vert _{W^{1,p}} \end{aligned}$$

from the uniform bi-Lipschitz property of $\sigma$ and the Sobolev embeddings. $\square$

### Existence of discrete-time approximations

After breaking the reparametrisation invariance of (1.1) by way of the approximate normal graphs, it is now a straight forward matter to prove the short-time existence of solutions for the minimising movement scheme.

Let us first consider an initial curve $\Gamma \in W^{2,p}({\mathbb {R}}/L{\mathbb {Z}},{\mathbb {R}}^n)$ of length *L* parametrised by arc-length. In the following, it will be essential that all estimates only depend on an upper bound on the energy of this curve.

We first note that an upper bound on the energy also implies a lower bound on the length, since by Fenchel’s theorem together with Hölder’s inequality we have

$$\begin{aligned} 2 \pi \le \int _{{\mathbb {R}}/ L {\mathbb {Z}}} |\kappa | \mathrm{d}s \le L ^{1-\frac{1}{p} } \bigg (\int _{{\mathbb {R}}/ L {\mathbb {Z}}} |\kappa |^p \mathrm{d}s \bigg )^{\frac{1}{p}} \end{aligned}$$

so that

$$\begin{aligned} L^ {p-1} \ge \frac{(2 \pi )^p}{p \, E^{(p)}(\Gamma )}. \end{aligned}$$

By scaling the results of Sect. 2.2, we can drop the assumption that the curve is of unit length and recover all the previous estimates concerning approximate normal graphs (with proviso that the relevant constants now depend on $\lambda$ and the energy bound). In particular, we say that the unit vector field $\tau$ is quasi-tangent to a $W^{2,p}$-curve $\gamma$ of length *L* whenever $\tau (\frac{\cdot }{L})$ is quasi-tangent to the curve $\gamma (\frac{\cdot }{L})$.

Now for the initial curve, the result of Lemma 2.12 implies that there exists a smooth curve $\widetilde{\gamma }$ parametrised by arc-length, a unit quasi-tangent $\tau$ to the curve $\widetilde{\gamma }$ and a perturbation $\Phi \in (W^{2,p})_\tau ^\perp$ such that $\Gamma \circ \sigma = \widetilde{\gamma }+ \Phi$. Moreover, by combining the norm bounds of Lemma 2.12 with Corollary 2.13 and the Sobolev embeddings, we see that

$$\begin{aligned} \quad \Vert \Phi \Vert _{L^\infty } \le \mu \quad \text {and}\quad \Vert \Phi '\Vert _{L^\infty } \le W \end{aligned}$$

for some sufficiently small constant $\mu =\mu (p,\lambda ,E(\Gamma ))>0$ and some constant $W = W (p,\lambda ,E(\Gamma ))>2$.

For a series of discrete time steps $0=t_0< t_1< t_2 < \cdots$, we seek to define the curves

2.11 $$\begin{aligned} \gamma _{t_j} = \widetilde{\gamma }+ \phi _{t_j} \end{aligned}$$

with the initial case $\gamma _{t_0} = \widetilde{\gamma }+ \Phi$. The time differences $t_{j+1} - t_j = h$ are set to be equal to a fixed parameter $h>0$ (that we shall ultimately send to zero). We want to recursively define $\phi _{t_{j+1}}$ for the next time step as the minimiser

$$\begin{aligned} \phi _{t_{j+1}} = \underset{\phi \,\in \, {\mathscr {V}}}{{{\,\mathrm{argmin}\,}}} \bigg \{ E(\widetilde{\gamma }+\phi ) + \frac{1}{2h} \int _{{\mathbb {R}} / L {\mathbb {Z}}} | P^{\bot }_{\gamma _{t_j}'}( \widetilde{\gamma }+ \phi - \gamma _{t_j}) |^2 |\gamma _{t_j}'|\mathrm{d}x \bigg \} , \end{aligned}$$

where the class of admissible perturbations is given by

$$\begin{aligned} {\mathscr {V}} = {\mathscr {V}}(\mu ,W) = \{\phi \in (W^{2,p})_\tau ^\bot : \Vert \phi \Vert _{L^\infty }< 3 \mu , \Vert \phi '\Vert _{L ^\infty } < 3 W\}. \end{aligned}$$

The following lemma states that these discrete-time solutions can be constructed for at least a short time.

#### Lemma 2.14

There exists a finite time $T>0$ depending only on *p*, $\lambda$ and $E(\Gamma )$ such that the solutions $\gamma _{t_j} = \widetilde{\gamma }+ \phi _{t_j}$ exist for the series of discrete times $0 = t_0< t_1< t_2< \cdots <t_N\le T$ where $N = \lfloor \tfrac{T}{h} \rfloor$.

#### Proof

We seek to establish the existence of the perturbations $\phi _{t_{j+1}}$ that are minimisers of the functionals

$$\begin{aligned} {\mathcal {F}}_j (\phi ) = E(\widetilde{\gamma }+\phi ) + \frac{1}{2h} \int _{{\mathbb {R}} / L {\mathbb {Z}}} | P^{\bot }_{\gamma _{t_j}'}( \widetilde{\gamma }+ \phi - \gamma _{t_j}) |^2 |\gamma _{t_j}'|\mathrm{d}x \end{aligned}$$

over the admissible class ${\mathscr {V}}$. To do so, we proceed by an induction argument with an initial base case $\phi _{t_0} = \Phi$ given by the decomposition of the initial curve $\Gamma$. Indeed, let us assume there exist minimisers $\phi _{t_{i+1}}$ of ${\mathcal {F}}_{i}$ over the class ${\mathscr {V}}$ for $i=0,1,\ldots ,j-1$.

Now as ${\mathcal {F}}_i (\phi _{t_{i+1}}) \le {\mathcal {F}}_i (\phi _{t_{i}})$ for $i=0,1,\ldots ,j-1$ (i.e. $\phi _{t_i}$ is a competitor), we note that

$$\begin{aligned} E(\gamma _{t_j}) \le E(\gamma _{t_0}) = E (\Gamma ) \end{aligned}$$

and

$$\begin{aligned} \frac{1}{2h} \int _{{\mathbb {R}} / L {\mathbb {Z}}} | P^{\bot }_{\gamma _{t_i}'}( \gamma _{t_{i+1}} - \gamma _{t_i}) |^2 |\gamma _{t_i}'|\mathrm{d}x \le E(\gamma _{t_i}) - E(\gamma _{t_{i+1}}). \end{aligned}$$

In which case Lemma 2.8 implies that the $L^p$-norm of $\gamma ''_{t_j}$ is uniformly bounded by a constant which depends only on *p* and $E(\Gamma )$. In addition, we have

$$\begin{aligned} \frac{1}{ h} \int _{{\mathbb {R}} /L {\mathbb {Z}}} | \gamma _{t_{i+1}} - \gamma _{t_i} |^2 \mathrm{d}x \le C \big ( E(\gamma _{t_i}) - E(\gamma _{t_{i+1}}) \big ) . \end{aligned}$$

Then by summing up the latter inequalities, we get the a priori estimate

2.12 $$\begin{aligned} \sum _{i=0}^{j-1} \frac{1}{ h} \int _{{\mathbb {R}} / L {\mathbb {Z}}} | \gamma _{t_{i+1}} - \gamma _{t_i} |^2 \mathrm{d}x \le C \big ( E(\gamma _{t_0}) - E(\gamma _{t_{j}}) \big ) .\end{aligned}$$

We also recall from Hölder’s inequality that

2.13 $$\begin{aligned} \Vert \gamma _{t_0} - \gamma _{t_{j}} \Vert _{L^2}\le & {} \sum _{i=0}^{j-1} \frac{\Vert \gamma _{t_{i+1}} - \gamma _{t_i} \Vert _{L^2} }{\sqrt{h}} \sqrt{h}\nonumber \\\le & {} \left( \sum _{i=0}^{j-1} \frac{1}{ h} \int _{{\mathbb {R}} / L {\mathbb {Z}}} | \gamma _{t_{i+1}} - \gamma _{t_i} |^2 \mathrm{d}x\right) ^{\frac{1}{2}} \left( \sum _{i=0}^{j-1} h \right) ^{\frac{1}{2} }\nonumber \\\le & {} C \sqrt{E(\gamma _{t_0})} \sqrt{t_{j} } \end{aligned}$$

and from the Gagliardo–Nirenberg interpolation inequality we get

$$\begin{aligned} \Vert \gamma _{t_0} ' - \gamma _{t_{j}} ' \Vert _{L^\infty } \le C \Vert \gamma _{t_0} '' - \gamma _{t_{j}} ''\Vert _{L^p}^\alpha \Vert \gamma _{t_0} - \gamma _{t_{j}} \Vert _{L^2}^{1-\alpha } \end{aligned}$$

with $\alpha = \frac{3p}{5p-2}$. Since Lemma 2.8 implies that the $L^p$-norm of the second derivatives of $\gamma _0$ and $\gamma _{t_j}$ are uniformly bounded, we conclude that

$$\begin{aligned} \Vert \gamma _{t_0} ' - \gamma _{t_{j}} ' \Vert _{L^\infty } \le C (\sqrt{t_j} ) ^{1-\alpha } \end{aligned}$$

for a constant $C>0$ depending on *p*, $\lambda$ and $E(\Gamma )$. Furthermore, there exists a sufficiently small $T>0$ depending on *p*, $\lambda$ and $E(\Gamma )$ such that

2.14 $$\begin{aligned} \Vert \phi _{t_j}'\Vert _{L^\infty }&\le \Vert \phi _{t_0}'\Vert _{L^\infty } + \Vert \gamma _{t_j} '- \gamma _{t_0} '\Vert _{L^\infty } \nonumber \\&\le W + C (\sqrt{t_j} ) ^{1-\alpha } \nonumber \\&< 2W \end{aligned}$$

whenever $$0< t_j<T$$. Since

$$\begin{aligned} \Vert \gamma _{t_0} - \gamma _{t_j}\Vert _{L^\infty } \le C \Vert \gamma _{t_0} '' - \gamma _{t_{j}} ''\Vert _{L^p}^\beta \Vert \gamma _{t_0} - \gamma _{t_{j}} \Vert _{L^2}^{1-\beta } \end{aligned}$$

with $\beta =\frac{p}{5p-2}$ by the Gagliardo–Nirenberg interpolation inequality, we also have

2.15 $$\begin{aligned} \Vert \phi _{t_j}\Vert _{L^\infty } < 2 \mu \end{aligned}$$

whenever $$0< t_j<T$$.

In fact, we can show that the same estimates hold for a suitably chosen minimising sequence. Let us assume that $(\phi _n)$ is a minimising sequence for the functional ${\mathcal {F}}_j$ in the class ${\mathscr {V}}$, i.e. ${\mathcal {F}}_j(\phi _n) \rightarrow \inf _{\phi \in {\mathscr {V}}} {\mathcal {F}}_j(\phi )$ and note that ${\mathcal {F}}_j$ is bounded from below by construction. As $\phi _{t_j}$ is still a competitor, we can assume without loss of generality that

$$\begin{aligned} {\mathcal {F}}_j( \phi _n) \le {\mathcal {F}}_j(\phi _{t_j}) = E(\gamma _{t_j}) \le E(\gamma _{t_0}) \end{aligned}$$

for all $n \in {\mathbb {N}}$. In which case we can repeat the argument from the above to obtain the bound

$$\begin{aligned} \Vert \gamma _{t_0} ' - \gamma _n' \Vert _{L^\infty } \le C ( \sqrt{t_{j+1}})^{1-\alpha } \end{aligned}$$

with $\gamma _n = \widetilde{\gamma }+ \phi _n$. It then follows that

2.16 $$\begin{aligned} \Vert \phi _{t_n}'\Vert _{L^\infty } < 2 W \end{aligned}$$

for all $0<t_{j+1}< T$. We then use the Gagliardo–Nirenberg interpolation inequality to obtain as above

2.17 $$\begin{aligned} \Vert \phi _{t_n}\Vert _{L^\infty } < 2 \mu . \end{aligned}$$

*Compactness*. As a consequence of Lemma 2.8, the minimising sequence $(\phi _n)$ is uniformly bounded in $W^{2,p}({\mathbb {R}}/L{\mathbb {Z}},{\mathbb {R}}^n)$. It then follows that there exists a weakly converging subsequence in $W^{2,p}({\mathbb {R}}/L{\mathbb {Z}},{\mathbb {R}}^n)$ which we also denoted by $(\phi _{n})$. In addition, the Rellich–Kondrašov compactness theorem implies that the subsequence $(\phi _{n})$ is strongly convergent in $C^1({\mathbb {R}}/L{\mathbb {Z}},{\mathbb {R}}^n)$. Let us denote the limit of this sequence by $\phi$. Since we have already established that $\Vert \phi _n\Vert _{L^\infty } < 2\mu$ and $\Vert \phi _n'\Vert _{L^\infty } < 2W$, it follows that $\Vert \phi \Vert _{L^\infty } \le 2\mu$ and $\Vert \phi '\Vert _{L^\infty } \le 2W$. Therefore, the limit $\phi$ also belongs to ${\mathscr {V}}$.

*Lower semi-continuity*. Let us finally prove that

$$\begin{aligned} {\mathcal {F}}_j (\phi ) \le \liminf _{n \rightarrow \infty } {\mathcal {F}}_j(\phi _n). \end{aligned}$$

As the $L^2$-term in the functional ${\mathcal {F}}_j$ converges by the theorem of Rellich–Kondrašov and the angle between $\tau$ and $\gamma _{t_j}'$ is uniformly bounded strictly away from $\tfrac{\pi }{2}$, it suffices to show that

2.18 $$\begin{aligned} E^{(p)} (\widetilde{\gamma }+ \phi ) \le \liminf _{n \rightarrow \infty } E^{(p)} (\widetilde{\gamma }+ \phi _n). \end{aligned}$$

Note that the length term $\lambda \int _{{\mathbb {R}}/L{\mathbb {Z}}} \mathrm{d}s$ appearing in the considered energy *E*, cf. (1.1), can be dropped as well due to the convergence of the sequence $(\phi _n)$ in $C^1({\mathbb {R}}/L{\mathbb {Z}},{\mathbb {R}}^n)$. In order to prove (2.18), we use the curvature formula for $\kappa _{{\widetilde{\gamma }} + \phi _n}$ to rewrite

$$\begin{aligned} E^{(p)} ( {\widetilde{\gamma }} + \phi _n) = \int _{{\mathbb {R}} /L {\mathbb {Z}}} \frac{ \left| P^\bot _{{\widetilde{\gamma }}' + \phi _n'} ({\widetilde{\gamma }}'' + \phi _n '') \right| ^p}{|{\widetilde{\gamma }}' + \phi _n'|^{2p}} |{\widetilde{\gamma }}' + \phi _n'| \mathrm{d}s \end{aligned}$$

as the expression

$$\begin{aligned} E^{(p)}( {\gamma }_n)&= \int _{{\mathbb {R}} / L {\mathbb {Z}}} \frac{ \left| P^\bot _{{\widetilde{\gamma }}' + \phi '} ({\widetilde{\gamma }}'' + \phi _n '') \right| ^p}{|{\widetilde{\gamma }}' + \phi '|^{2p}} |{\widetilde{\gamma }}' + \phi '| \mathrm{d}s + {\mathscr {I}}_1 + {\mathscr {I}}_2 + {\mathscr {I}}_3,\end{aligned}$$

where

$$\begin{aligned} {\mathscr {I}}_1&= \int _{{\mathbb {R}} /L {\mathbb {Z}}} \bigg ( { \big |P^\bot _{{\widetilde{\gamma }}' + \phi _n'} ({\widetilde{\gamma }}'' + \phi _n '') \big |^p} - { \big |P^\bot _{{\widetilde{\gamma }}' + \phi '} ({\widetilde{\gamma }}'' + \phi _n '') \big |^p} \bigg ) \frac{|{\widetilde{\gamma }}' + \phi _n'|}{|{\widetilde{\gamma }}' + \phi _n'|^{2p}} \mathrm{d}s, \\ {\mathscr {I}}_2&=\int _{{\mathbb {R}} /L {\mathbb {Z}}} \left( \frac{1}{|{\widetilde{\gamma }}' + \phi _n'|^{2p}} - \frac{1}{|{\widetilde{\gamma }}' + \phi '|^{2p}} \right) \left| P^\bot _{{\widetilde{\gamma }}' + \phi '} ({\widetilde{\gamma }}'' + \phi _n '') \right| ^p |{\widetilde{\gamma }}' + \phi _n'| \mathrm{d}s, \\ {\mathscr {I}}_3&=\int _{{\mathbb {R}} / L {\mathbb {Z}}} \frac{ \left| P^\bot _{{\widetilde{\gamma }}' + \phi '} ({\widetilde{\gamma }}'' + \phi _n '') \right| ^p}{|{\widetilde{\gamma }}' + \phi '|^{2p}} \bigg ( |{\widetilde{\gamma }}' + \phi _n'| - |{\widetilde{\gamma }}' + \phi '|\bigg ) \mathrm{d}s .\end{aligned}$$

The terms ${\mathscr {I}}_1$, ${\mathscr {I}}_2$ and ${\mathscr {I}}_3$ vanish in the limit due to the convergence of the sequence $(\phi _n)$ in $C^1({\mathbb {R}}/L{\mathbb {Z}},{\mathbb {R}}^n)$ and the uniform bound on the $W^{2,p}$-norm of $\phi _n$. Moreover, the expression

$$\begin{aligned} {\mathscr {I}}({\widetilde{\gamma }} + \phi _n) = \int _{{\mathbb {R}} / L {\mathbb {Z}}} \frac{ \left| P^\bot _{{\widetilde{\gamma }}' + \phi '} ({\widetilde{\gamma }}'' + \phi _n '') \right| ^p}{|{\widetilde{\gamma }}' + \phi '|^{2p}} |{\widetilde{\gamma }}' + \phi '| \mathrm{d}s \end{aligned}$$

is convex and continuous on $W^{2,p}({\mathbb {R}} / L {\mathbb {Z}}, {\mathbb {R}}^n)$ and hence lower semi-continuous by the following standard argument: Mazur’s lemma [25, Theorem 3.13] gives for every $n_0 \in {\mathbb {N}}$ a sequence of convex combinations

$$\begin{aligned} P^l = \sum _{n=n_0}^l \alpha _n^l\phi _n , \quad 0 \le \alpha _n^l \le 1, \quad \sum _{n=n_0}^l \alpha _n^l =1 \end{aligned}$$

such that $P^l\rightarrow \phi$ strongly in $W^{2,p}({\mathbb {R}} / L {\mathbb {Z}}, {\mathbb {R}}^n).$ The convexity of ${\mathscr {I}}$ now implies

$$\begin{aligned} {\mathscr {I}} (\widetilde{\gamma }+P^l) = {\mathscr {I}} ( \sum _{n=n_0}^l \alpha _n^l ( \widetilde{\gamma }+\phi _n )) \le \sum _{n=n_0}^l \alpha _n^l {\mathscr {I}} (\widetilde{\gamma }+ \phi _n ) \le \sup _{n \ge n_0} {\mathscr {I}} (\widetilde{\gamma }+\phi _n ). \end{aligned}$$

Passing to the limit $l \rightarrow 0$ on the left-hand side, we get from the continuity of ${\mathscr {I}}$ and $P^l \rightarrow \phi$ in $W^{2,p}$ that

$$\begin{aligned} {\mathscr {I}} (\widetilde{\gamma }+\phi ) \le \sup _{n \ge n_0} {\mathscr {I}} (\widetilde{\gamma }+\phi _n ) \end{aligned}$$

for all $n_0 \in {\mathbb {N}}$. This yields

$$\begin{aligned} {\mathscr {I}} (\widetilde{\gamma }+\phi ) \le \inf _{n_0 \in {\mathbb {N}}} \sup _{n \ge n_0} {\mathscr {I}} (\widetilde{\gamma }+\phi _n ) = \liminf _{n \rightarrow \infty } {\mathscr {I}} (\widetilde{\gamma }+\phi _n ). \end{aligned}$$

$$\square $$

For later reference, let us also state the following a priori estimate for the piecewise linear interpolations that results from (2.12) and (2.13).

#### Corollary 2.15

The piecewise linear interpolations

$$\begin{aligned} \phi ^{(h)}(t, \,\cdot \,) = \phi _{t_{j}} + \frac{t-t_j}{h} \big (\phi _{t_{j+1}} - \phi _{t_j} \big ) , \quad t_j \le t \le t_{j+1}, \end{aligned}$$

satisfies the estimates

$$\begin{aligned} \Vert \phi _{t''}^{(h)} - \phi _{t'}^{(h)} \Vert _{L^2} \le C \sqrt{t'' - t'} \end{aligned}$$

and

$$\begin{aligned} \int _{t'}^{t''} \int _{{\mathbb {R}} /L {\mathbb {Z}}} |\partial _t \phi ^{(h)}(t,s) |^2 \mathrm{d}s \mathrm{d}t \le C \big (E(\gamma _{t'}) - E(\gamma _{t''}) \big ) \end{aligned}$$

for any $$0\le t'< t''< T<\infty $$.

#### Remark 2.16

We thus obtain a piecewise linearly interpolated solution

2.19 $$\begin{aligned} \gamma _t^{(h)} = \widetilde{\gamma }+ \phi ^{(h)}_t, \quad 0\le t < T, \end{aligned}$$

for the minimising movements scheme.

## Weak solutions

### Euler–Lagrange equations for the approximations

In order to improve the regularity of the approximations, we derive the Euler–Lagrange equations related to the minimising movement scheme.

We recall the following expression (cf. [9, Lemma 2.1]) for the first variation of the *p*-elastic energy, namely

3.1 $$\begin{aligned} \delta _\psi E^{(p)} (\gamma ) = \int _{{\mathbb {R}} /L {\mathbb {Z}}} |\kappa |^{p-2} \langle \kappa , \delta _\psi \kappa \rangle \mathrm{d}s + \frac{1}{p} \int _{{\mathbb {R}} / L {\mathbb {Z}}} |\kappa |^p \langle \partial _s \gamma , \partial _s \psi \rangle \mathrm{d}s \end{aligned}$$

where $\delta _\psi \kappa = \left( \partial _s^2 \psi \right) ^\bot - \langle \kappa , \partial _s \psi \rangle \partial _s \gamma - 2\langle \partial _s \gamma , \partial _s \psi \rangle \kappa$, cf. Proposition A.1. The first variation of the length term appearing in the definition of the energy *E*, cf. (1.1), is given by

3.2 $$\begin{aligned} \delta _\psi \Big ( \lambda \int _{{\mathbb {R}}/L{\mathbb {Z}}} ds\Big ) = \lambda \int _{{\mathbb {R}}/L{\mathbb {Z}}} \langle \partial _s\gamma , \partial _s \psi \rangle \mathrm{d}s. \end{aligned}$$

Combining (3.1) and (3.2) with the fact that

$$\begin{aligned} \partial _s \psi = \frac{1}{|\gamma '|} \partial _x \psi , \end{aligned}$$

where $|\gamma '|=|\partial _x \gamma |$, we get

$$\begin{aligned} \partial ^2 _s \psi&= \frac{1}{|\gamma '|} \partial _x \Big ( \frac{1}{|\gamma '|} \partial _x \psi \Big ) = \frac{1}{|\gamma '|^2} \partial _x ^2 \psi - \frac{1}{|\gamma '| ^3 } \Big \langle \frac{\gamma '}{|\gamma '|} , \gamma '' \Big \rangle \partial _x \psi \end{aligned}$$

so that

$$\begin{aligned} \delta _\psi E (\gamma ) = \int _{{\mathbb {R}} /L {\mathbb {Z}}} \frac{|\kappa |^{p-2} }{|\gamma '|} \langle \kappa , \partial _x^2 \psi \rangle \mathrm{d}x + R(\psi ) , \end{aligned}$$

where $R(\psi )$ has the form

$$\begin{aligned} R(\psi ) = \int _{{\mathbb {R}} / L{\mathbb {Z}}} \langle b , \partial _x \psi \rangle \mathrm{d}x \end{aligned}$$

for

$$\begin{aligned} b = \frac{|\kappa |^{p-2}}{|\gamma '|^3} \langle \gamma ', \gamma '' \rangle \kappa - |\kappa |^{p-2} \langle \kappa , \gamma ' \rangle \kappa - (2-\frac{1}{p}) |\kappa |^{p} \gamma '. \end{aligned}$$

Note that $b \in L^{\infty }L^{1}$ for any time-dependent family of curves $\gamma \in L^\infty W^{2,p}$, whereas a notational shorthand $L^{\infty }L^{1}$ stands for $L^\infty \big ([0,T), L^{1}({\mathbb {R}} / {\mathbb {Z}}, {\mathbb {R}}^n)\big )$.

On the other hand, solutions of the minimising movement scheme solve

3.3 $$\begin{aligned} \langle \partial _t \gamma , P_\tau ^\bot \psi \rangle = - \delta _\psi E (\gamma ) \end{aligned}$$

for all $\psi \in \left( W^{2,p} \right) ^\bot _\tau$. Therefore, we conclude that

3.4 $$\begin{aligned} \int _{{\mathbb {R}} / L {\mathbb {Z}}} \frac{|\kappa |^{p-2} }{|\gamma '|} \langle \kappa , \partial _x^2 \psi \rangle \mathrm{d}x + \widetilde{R}(\psi ) = 0 , \end{aligned}$$

where

$$\begin{aligned} \widetilde{R}(\psi ) = \int _{{\mathbb {R}} / L {\mathbb {Z}}} \langle b, \partial _x \psi \rangle \mathrm{d}x +\int _{{\mathbb {R}} / L {\mathbb {Z}}} \langle P_\tau ^\bot (\partial _t \gamma ) , \psi \rangle \mathrm{d}x . \end{aligned}$$

### Higher regularity for the approximations

To deduce regularity from the equation above, we consider a smooth local orthonormal basis $\nu _1,\ldots ,\nu _{n-1}$ for our approximate normal spaces. If $\psi$ is a test function that is decomposed into the form

$$\begin{aligned} \psi = \sum _{i=1}^{n-1} \psi _i \nu _i \end{aligned}$$

such that the scalar functions $\psi _i$ vanish away from the neighbourhood, we find that

$$\begin{aligned} \partial ^2_x \psi = \sum _{i=1}^{n-1} \Big ( \partial _x^2 \psi _i \nu _i + 2 \partial _x \psi _i \partial _x \nu _i + \psi _i \partial _x^2 \nu _i \Big ). \end{aligned}$$

Therefore, the evolution equation for the approximation yields

3.5 $$\begin{aligned} \sum _{i=1}^{n-1} \int _{{\mathbb {R}} / L {\mathbb {Z}}} \frac{|\kappa |^{p-2} }{|\gamma '|} \partial _x^2 \psi _i \langle \kappa , P_\tau ^\bot \nu _i \rangle \mathrm{d}x = Q(h) , \end{aligned}$$

where

$$\begin{aligned} Q(h) = \int _{{\mathbb {R}} / L {\mathbb {Z}}} \langle b_t , \partial _x \psi \rangle + \langle c_t , \psi \rangle +\langle P_\tau ^\bot (\partial _t \gamma (t, \,\cdot \,)) ,\psi \rangle \mathrm{d}x. \end{aligned}$$

The following lemma helps us to deduce regularity from this form of the equation.

#### Lemma 3.1

($L^1$-estimates) Let $I = (a,b)$ be an open subset of ${\mathbb {R}}$. If there exist functions *u*, *f* and *F* in $L^1 (I)$ such that

$$\begin{aligned} \int _I \left( u \partial _x^2 \varphi + F \partial _x \varphi \right) \mathrm{d}x = \int _I f \varphi \mathrm{d}x \end{aligned}$$

for all $\varphi \in C^{\infty }_c (I)$, then

$$\begin{aligned} u(x) =\int _a^x \left( F(y) + \int _a^y f(z) \mathrm{d}z \right) dy+m (x-a) + d \end{aligned}$$

with $$d = \lim _{x \searrow a} u(x)$$ and

$$\begin{aligned} m(b-a) = \lim _{x \nearrow b} u(x) - \left( \int _I \left( F(y) + \int _a^y f(z) \mathrm{d}z \right) \mathrm{d}y + d\right) . \end{aligned}$$

Moreover, the function $u \in W^{1,1}(I)$ with

$$\begin{aligned} \Vert u\Vert _{W^{1,1}} \le C (\Vert f\Vert _{L^1} + \Vert F\Vert _{L^1}). \end{aligned}$$

#### Proof

Let us first set

$$\begin{aligned} w(x)&= F(x) + \int _a^x f(y) \mathrm{d}y \\ v(x)&= \int _a^x w(y) \mathrm{d}y \end{aligned}$$

and note that $v \in W^{1,1} (I)$ with $v'=w$. Then integration by parts implies that

$$\begin{aligned} \int _I v(x) \partial _x ^2 \varphi (x) \, \mathrm{d}x&= - \int _{I} v'(x) \partial _x \varphi (x) \, \mathrm{d}x \\&= - \int _{I} \left( F(x) \partial _x \varphi (x) + \Big ( \int _a^x f(y) \mathrm{d}y \Big ) \partial _x \varphi (x) \right) \mathrm{d}x \\&= -\int _I \left( F(x) \partial _x \varphi (x) - f(x) \varphi (x) \right) \mathrm{d}x .\end{aligned}$$

Therefore,

$$\begin{aligned} \int _I (u-v) \partial _x^2 \varphi \,\mathrm{d}x =0 \end{aligned}$$

for all $\varphi \in C^\infty _c (I)$. In which case $u-v$ is an affine function from which the conclusion easily follows. $\square$

We can now use the latter lemma to establish:

#### Theorem 3.2

(Higher regularity) If $\gamma _t^{(h)}$ is a solution to the minimising movements scheme given by (2.19), there exists a constant $C>0$ independent of *h* such that

3.6 $$\begin{aligned} \left\| |\kappa |^{p-2} P^\bot _\tau \kappa \right\| _{L^2([0,T), W^{1,1}) } \le C . \end{aligned}$$

In particular, we have $\kappa$ uniformly bounded in $L^2L^q$ and $\gamma '$ uniformly bounded in $L^2([0,T),W^{1,q})$ for all $1\le q<\infty$.

#### Proof

This higher regularity result directly follows from the application of Lemma 3.1 to our evolution equation for the minimising movement scheme approximations. In particular, from Corollary 2.15 we see that $\gamma _t^{(h)}$ satisfies

$$\begin{aligned} \int _{0}^T \int _{{\mathbb {R}} / L {\mathbb {Z}}} |\partial _t \gamma ^{(h)} (t,s)|^2 \mathrm{d}s \mathrm{d}t \le C E(\gamma _0). \end{aligned}$$

Applying Lemma 2.8 to (3.5) together with a covering argument hence yields

$$\begin{aligned} \Big \Vert \frac{|\kappa |^{p-2}}{|(\gamma ^{(h)})'|} P^{\bot }_\tau \kappa \Big \Vert _{L^2([0,T), W^{1,1}) } < C. \end{aligned}$$

Since $(\gamma ^{(h)})'$ is uniformly bounded in $W^{1,1}$ and $W^{1,1}$ is a Banach algebra, this implies

$$\begin{aligned} \Vert |\kappa |^{p-2}P^{\bot }_\tau \kappa \Vert _{L^2([0,T), W^{1,1}) } < C. \end{aligned}$$

$$\square $$

### Convergence to weak solutions

We will use the following result in order to obtain the convergence of solutions. This result is crucial for the control of the terms involving the energy.

#### Theorem 3.3

Let $\gamma _n = \gamma + \phi _n$ be a sequence bounded in $L^\infty W^{2,p} \cap C^{\frac{1}{2}}L^2$ such that $|\kappa _n|^{p-2}\kappa _n$ is uniformly bounded in $L^2W^{1,1}$. Then there exists a subsequence $\gamma _{n_j}$ such that the curvatures $\kappa _{n_j}$ converge in $L^2W^{2,p}$.

The proof of this theorem relies on the following interpolation estimate.

#### Lemma 3.4

There exists a constant $C_0>0$ depending on *p* such that for any $W^{2,p}$-curves $\gamma _1$ and $\gamma _2$ with curvatures $\kappa _1$ and $\kappa _2$ we have

$$\begin{aligned} \Vert \kappa _1 - \kappa _2\Vert _{L^p} \le C_0 (\Vert |\kappa _1|^{p-2} \kappa _1\Vert _{L^2 W^{1,1}} + \Vert |\kappa _2|^{p-2}\kappa _2\Vert _{L^2W^{1,1}}) \Vert \gamma '_1 - \gamma '_2\Vert _{L^2L^\infty }. \end{aligned}$$

If these curves are furthermore approximate normal graphs over $\widetilde{\gamma }$ as for the solutions to the minimising movement scheme, we get

$$\begin{aligned}&\Vert \kappa _1 - \kappa _2\Vert _{L^p} \\&\le C_0 (\Vert |\kappa _1|^{p-2} P^\bot _\tau \kappa _1\Vert _{L^2 W^{1,1}} + \Vert |\kappa _2|^{p-2} P^\bot _\tau \kappa _2\Vert _{L^2W^{1,1}}) \Vert \gamma '_1 - \gamma '_2\Vert _{L^2L^\infty } \end{aligned}$$

where now $$C=C(\lambda , p, E(\Gamma )).$$

#### Proof

First note that

$$\begin{aligned} \int |\kappa _1 - \kappa _2|^{p} \mathrm{d}s \le C_0\int \left( |\kappa _1|^{p-2} \kappa _1 - |\kappa _2|^{p-2} \kappa _2 \right) (\kappa _1 - \kappa _2) \mathrm{d}s \end{aligned}$$

(cf. [8, §1, Lemma 4.4]). Then integration by parts and Hölder’s inequality implies that

$$\begin{aligned} \int |\kappa _1 - \kappa _2|^{p} \mathrm{d}s&\le - C_0\int \partial _s \left( |\kappa _1|^{p-2} \kappa _1 - |\kappa _2|^{p-2} \kappa _2 \right) (\partial _s \gamma _1 - \partial _s \gamma _2) \mathrm{d}s \\&\le C_0 \left( \left\| |\kappa _1|^{p-2} \kappa _1 \right\| _{W^{1,1}} + \left\| |\kappa _2|^{p-2} \kappa _2 \right\| _{W^{1,1}} \right) \Vert \gamma _1' - \gamma _2 '\Vert _{L^{\infty }}. \end{aligned}$$

So by integrating over time and using Hölder’s inequality again, we get

$$\begin{aligned}&\iint |\kappa _1 - \kappa _2|^{p} ds dt \\&\qquad \le C_0 \left( \left\| |\kappa _1|^{p-2} \kappa _1 \right\| _{L^2W^{1,1}} + \left\| |\kappa _2|^{p-2} \kappa _2 \right\| _{L^2W^{1,1}} \right) \Vert \gamma _1' - \gamma _2 '\Vert _{L^2L^{\infty }} . \end{aligned}$$

For the second estimate, we proceed in a similar way. We apply Lemma 2.8 to improve the first inequality to

$$\begin{aligned} \int |\kappa _1 - \kappa _2|^{p} \mathrm{d}s \le C_0\int \left( |\kappa _1|^{p-2} P^\bot _\tau \kappa _1 - |\kappa _2|^{p-2} P^\bot _\tau \kappa _2 \right) (\kappa _1 - \kappa _2) \mathrm{d}s. \end{aligned}$$

Integrating by parts then yields

$$\begin{aligned}&\int |\kappa _1 - \kappa _2|^{p} \mathrm{d}s \le - C_0\int \partial _s \left( |\kappa _1|^{p-2} P^\bot _\tau \kappa _1 - |\kappa _2|^{p-2} P^\bot _\tau \kappa _2 \right) (\partial _s \gamma _1 - \partial _s \gamma _2) \mathrm{d}s\\&\le C_0 \left( \left\| |\kappa _1|^{p-2} P^\bot \kappa _1 \right\| _{W^{1,1}} + \left\| |\kappa _2|^{p-2} P^\bot _{\tau }\kappa _2 \right\| _{W^{1,1}} \right) \Vert \gamma _1' - \gamma _2 '\Vert _{L^{\infty }}. \end{aligned}$$

$$\square $$

#### Proof of Theorem 3.3

Using a diagonal argument and the compact embedding $W^{2,p} \hookrightarrow L^2$, we get a subsequence $\gamma _{n_j}$ converging in $L^2$ for all times $t \in {\mathbb {Q}} \cap [0,T)$ (and hence for all $0\le t <T$ due to the uniform bound in $C^{\frac{1}{2}} L^2$). This result, together with the uniform bound on the $W^{2,p}$-Sobolev norm and interpolation estimates, implies that $\gamma _{n_j} \rightarrow \gamma \in C^{\alpha } ([0,T), W^{1,\infty })$ with $\alpha = \frac{p-1}{5p-2}$. Thus, $\gamma _{n_j}$ converge to $\gamma$ in $L^2W^{2,p}$ by Lemma 3.4. $\square$

#### Proof of Theorem 1.1

From the construction in Sect. 2.3, there exists a solution $\gamma ^{(h)}_t$ to the minimising movement scheme given by (2.19) for all $0\le t <T$ up to some positive final time *T* that depends only on *p*, $\lambda$ and the energy $E(\Gamma )$ of the initial data. We think of this solution as solving a discrete version of the negative $L^2$-gradient flow of *E*. Theorem 3.3 and Corollary 2.15 can then be applied to get a subsequence that converges in $L^2W^{2,p}$ such that $\partial _t \gamma ^{(h)}$ weakly converges in $L^2$. Now in order to show that the limit satisfies the desired evolution equations, we use the fact that the solutions of the minimising movement scheme satisfy

3.7 $$\begin{aligned} \int _0^T \int _{{\mathbb {R}} / L {\mathbb {Z}}} \langle \partial _t ^\bot \gamma _t^{(h)} , \psi \rangle \mathrm{d}s \mathrm{d}t = \int _{{\mathbb {R}} / L {\mathbb {Z}}} \delta _{\psi _t} E ( \widetilde{\gamma }^{(h)}_t) \mathrm{d}t \end{aligned}$$

for all test functions $\psi \in C_c^\infty ((0,T) \times {\mathbb {R}} / L {\mathbb {Z}} ,{\mathbb {R}}^n)$. Here, $\widetilde{\gamma }^{(h)}_t = \gamma ^{(h)}_{nh}$ for $t\in [nh, (n+1)h)$, $n \in {\mathbb {N}}$, denotes the piecewise constant interpolation of the minimising movement scheme.

Let us now take a sequence $h_n \rightarrow 0$ for which the piecewise linear interpolations of the minimising movement scheme $\gamma ^{(h_n)}$ converge to a family of curves $\gamma$ in $L^2W^{2,p}$ such that $\partial _t \gamma ^{(h_n)}$ converges to $\partial _t \gamma$ weakly in $L^2([0,T), {\mathbb {R}} / L {\mathbb {Z}})$. Then also the piecewise constant interpolations $\widetilde{\gamma }^{(h_n)}$ converge to $\gamma$ in $L^2W^{2,p}$. As $\gamma '^{(h_n)}$ converges strongly to $\gamma '$ in $L^2$, we see that the weak convergence of $\partial _t \gamma ^{(h_n)}$ to $\partial _t \gamma$ in $L^2$ implies

3.8 $$\begin{aligned} \int _0^T \int _{{\mathbb {R}} / L{\mathbb {Z}}} \langle \partial _t ^\bot \gamma ^{(h_n)}_t , \psi \rangle \mathrm{d}s \mathrm{d}t \rightarrow \int _0^T \int _{{\mathbb {R}} /L {\mathbb {Z}}} \langle \partial _t ^\bot \gamma _t , \psi \rangle \mathrm{d}s \mathrm{d}t. \end{aligned}$$

Convergence for the right-hand side of (3.7) is also straight forward. If we denote by $\kappa _n$ the curvature of $\widetilde{\gamma }^{(h_n)}_t$ and integrate (3.1), we find that

$$\begin{aligned} \int _0^T \delta _{\psi _t} E (\widetilde{\gamma }^{(h_n)}_t ) \mathrm{d}t&= \int _0^T \int _{{\mathbb {R}} / L {\mathbb {Z}}} |\kappa _n|^{p-2} \langle \kappa _n , \delta _\psi \kappa _n \rangle \mathrm{d}s \mathrm{d}t \\&\qquad + \frac{1}{p} \int _0^T \int _{{\mathbb {R}} / L {\mathbb {Z}}} |\kappa _n|^p \langle \partial _s \widetilde{\gamma }^{(h_n)}, \partial _s \psi \rangle \mathrm{d}s \mathrm{d}t \\&\qquad + \lambda \int _0^T \int _{{\mathbb {R}} /L {\mathbb {Z}}} \langle \partial _s \widetilde{\gamma }^{(h_n)}, \partial _s \psi \rangle \mathrm{d}s \mathrm{d}t . \end{aligned}$$

Since $\kappa _n$ converges to $\kappa$ in $L^2([0,T), L^p( {\mathbb {R}} / L {\mathbb {Z}} ))$ and $\partial _s \widetilde{\gamma }^{(h_n)}$ converges to $\partial _s\gamma$ uniformly, the second term on the right-hand side of the latter equation converges to the corresponding term for $\gamma$ in lieu of $\widetilde{\gamma }^{(h_n)}$. One can deduce the same fact for the first term via the formula

$$\begin{aligned} \delta _\psi \kappa _n = \left( \partial _s^2 \psi \right) ^\bot - \langle \kappa _n , \partial _s \psi \rangle \tau _n -\langle \partial _s \psi , \tau _n \rangle \kappa _n , \end{aligned}$$

since it implies that $\delta _\psi \kappa _n$ converges to $\delta _\psi \kappa$ in $L^2 ([0,T), L^p( {\mathbb {R}} /L {\mathbb {Z}} ))$. Therefore, we get

3.9 $$\begin{aligned} \int _{0}^T \delta _{\psi _t} E (\widetilde{\gamma }^{(h_n)}) \mathrm{d}t \rightarrow \int _{0}^T \delta _{\psi _t} E (\gamma ) \mathrm{d}t . \end{aligned}$$

In which case Eqs. (3.7), (3.8) and (3.9) imply that

$$\begin{aligned} \int _0^T \int _{{\mathbb {R}} / L {\mathbb {Z}}} \langle \gamma _t ,\partial _t ^\bot \psi \rangle \mathrm{d}s \mathrm{d}t = - \int _0^T \delta _{\psi _t} E (\gamma _t)\mathrm{d}t. \end{aligned}$$

$$\square $$

### Flow in the direction of the normal velocity

Using the fact that the unit tangent belongs to $W^{2,p}$, we can finally prove Corollary 1.2 under the conditions of Theorem 1.1.

#### Proof of Corollary 1.2

In abuse of notation, let $\tau = \frac{\gamma '}{|\gamma '|} \in W^{2,p}({\mathbb {R}} / L {\mathbb {Z}}, {\mathbb {R}}^n)$ be the unit tangent and the vectors $\nu _1, \ldots , \nu _{n-1}$ be a smooth local orthonormal basis of our approximate normal space. Due to the fact that any $\psi \in C_c^{\infty }({\mathbb {R}} / L {\mathbb {Z}}, {\mathbb {R}}^n)$ can be written as

$$\begin{aligned} \psi = \psi _0 \tau + \sum _{i=1}^{n-1} \psi _i \nu _i \end{aligned}$$

with functions $\psi _i \in W^{2,p}({\mathbb {R}} /L {\mathbb {Z}}, {\mathbb {R}}^n)$, we find that

$$\begin{aligned} \int _0^T \int _{{\mathbb {R}} / L {\mathbb {Z}}} \langle \partial ^\bot _t \gamma ,\psi \rangle \mathrm{d}s \mathrm{d}t&= \sum _{i=1}^{n-1} \int _0^T \int _{{\mathbb {R}} /L {\mathbb {Z}}} \langle \partial ^\bot _t \gamma , \psi _i \nu _i \rangle \mathrm{d}s \mathrm{d}t \\&= -\int _0^T \delta _{\psi _t} E(\gamma _t) \mathrm{d}t , \end{aligned}$$

since both $\delta _{\psi _0 \tau } E (\gamma ) =0$ and $\langle \partial _t^\bot \psi _0, \tau \rangle =0$. $\square$

## Epilogue

Although the minimising movement scheme leads in a rather straight forward way to the short-time existence of weak solution for our gradient flow, there are three key questions one would like to resolve, namely:

1. Are weak solutions unique and do they have long-time existence for $0\le t<\infty$?
2. Can one use test functions for the gradient flow that are not orthogonal to a quasi-tangent?
3. Does our notion of solution depend on the choice of the reference curve and the approximate normal directions?

For long-time existence, it looks as if one could, in principle, restart the flow and the above short-time existence result to get an eternal solution. However, one should be aware that this solution might have kinks which our methods cannot rule out. If one has uniqueness and some way of modifying the approximate normal, long-time existence would be possible. Our Corollary 1.2 is a first indication that a more fastidious regularity theory is needed in order to resolve the above issues.

The question of uniqueness seems to be completely open. For the more standard non-homogeneous evolution equations involving the *p*-Laplace operator, papers discussing uniqueness have only appeared rather recently. In particularly, the method used to prove uniqueness in [3] breaks down for our curvature equations.

## Appendix A: First variation for the *p*-elastic energy

Recall that for closed curves $\gamma :{\mathbb {R}} / {\mathbb {Z}} \rightarrow {\mathbb {R}}^n$ in the $W^{2,p}$-Sobolev class the *p*-elastic energy is given by

$$\begin{aligned} E^{(p)}(\gamma ) = \tfrac{1}{p} \int _{{\mathbb {R}}/ {\mathbb {Z}}} |\kappa |^p \mathrm{d}s. \end{aligned}$$

For the convenience of the reader, we give further details on the derivation of its first variation. The upcoming statement is proven along the line of [9, Lemma 2.1], for which we identify the arclength element by $ds = |\partial _x \gamma | \, dx$ and the arclength derivative by $\partial _s = |\partial _x \gamma |^{-1} \partial _x$.

### Proposition A.1

The first variation of the *p*-elastic energy $E^{(p)}$ for $\gamma \in W^{2,p}({\mathbb {R}}/{\mathbb {Z}},{\mathbb {R}}^n)$ in direction of $\psi \in W^{2,p}({\mathbb {R}}/{\mathbb {Z}},{\mathbb {R}}^n)$ is given by

$$\begin{aligned} \delta _\psi E^{(p)} (\gamma ) = \int _{{\mathbb {R}} / {\mathbb {Z}}} |\kappa |^{p-2} \langle \kappa , \delta _\psi \kappa \rangle \mathrm{d}s + \tfrac{1}{p} \int _{{\mathbb {R}} / {\mathbb {Z}}} |\kappa |^p \langle \partial _s \gamma , \partial _s \psi \rangle ds. \end{aligned}$$

where $$ \delta _\psi \kappa = \left( \partial _s^2 \psi \right) ^\bot - \langle \kappa , \partial _s \psi \rangle \partial _s \gamma - 2\langle \partial _s \gamma , \partial _s \psi \rangle \kappa $$.

### Proof

We first observe

$$\begin{aligned} \delta _\psi E^{(p)} (\gamma ) = \tfrac{1}{p} \int _{{\mathbb {R}} / {\mathbb {Z}}} \delta _\psi (|\kappa |^{p} ) \mathrm{d}s + \tfrac{1}{p} \int _{{\mathbb {R}} / {\mathbb {Z}}} |\kappa |^p \delta _\psi ( \mathrm{d}s). \end{aligned}$$

By applying the notation from above and the chain rule, we get

$$\begin{aligned} \delta _\psi (|\kappa |^{p} )&=\tfrac{d}{d\varepsilon } \left[ |\partial _s^2 (\gamma +\varepsilon \psi )|^{p} \right] _{\varepsilon =0} \\&= \left[ p \, |\langle \partial _s^2(\gamma +\varepsilon \psi ), \partial _s^2(\gamma + \varepsilon \psi )\rangle |^{\frac{p-2}{2}} \langle \tfrac{d}{d\varepsilon } \partial _s^2(\gamma +\varepsilon \psi ), \partial _s^2(\gamma +\varepsilon \psi ) \rangle \right] _{\varepsilon =0} \\&= p \, |\kappa |^{p-2} \langle \delta _\psi (\kappa ), \kappa \rangle \end{aligned}$$

and

$$\begin{aligned} \delta _\psi (ds)&= \tfrac{d}{d\varepsilon }\left[ |\partial _x(\gamma +\varepsilon \psi )| dx\right] _{\varepsilon =0} \\&= \left[ \tfrac{1}{|\partial _x(\gamma +\varepsilon \psi )|} \langle \partial _x (\gamma + \varepsilon \psi ), \partial _x \psi \rangle dx \right] _{\varepsilon =0} \\&= \langle \tfrac{\partial _x \gamma }{|\partial _x \gamma |},\tfrac{\partial _x \psi }{|\partial _x \gamma |}\rangle |\partial _x \gamma | dx \\&= \langle \partial _s \gamma , \partial _s \psi \rangle ds. \end{aligned}$$

Similarly, we achieve

$$\begin{aligned}&\delta _\psi (\kappa ) =\tfrac{d}{d\varepsilon } \left[ \tfrac{1}{|\partial _x(\gamma + \varepsilon \psi )|} \partial _x \left( \tfrac{1}{|\partial _x(\gamma +\varepsilon \psi )|} \partial _x (\gamma + \varepsilon \psi )\right) \right] _{\varepsilon =0} \\&= \left[ - \tfrac{1}{|\partial _x(\gamma + \varepsilon \psi )|^3} \langle \partial _x(\gamma + \varepsilon \psi ), \partial _x \psi \rangle \partial _x \left( \tfrac{1}{|\partial _x(\gamma +\varepsilon \psi )|} \partial _x (\gamma + \varepsilon \psi )\right) \right] _{\varepsilon =0} \\&\quad + \left[ \tfrac{1}{|\partial _x(\gamma + \varepsilon \psi )|} \partial _x (-\tfrac{1}{|\partial _x(\gamma +\varepsilon \psi )|^3} \langle \partial _x(\gamma +\varepsilon \psi ),\partial _x h \rangle \partial _x (\gamma + \varepsilon \psi ) + \tfrac{1}{|\partial _x(\gamma +\varepsilon \psi )|} \partial _x h) \right] _{\varepsilon =0} \\&= - \langle \tfrac{\partial _x \gamma }{|\partial _x\gamma |}, \tfrac{\partial _x \psi }{|\partial _x\gamma |} \rangle \tfrac{1}{|\partial _x\gamma |} \partial _x (\tfrac{\partial _x \gamma }{|\partial _x\gamma |}) - \tfrac{1}{|\partial _x\gamma |} \partial _x \left( \langle \tfrac{\partial _x \gamma }{|\partial _x\gamma |}, \tfrac{\partial _x \psi }{|\partial _x\gamma |} \rangle \tfrac{\partial _x \gamma }{|\partial _x\gamma |} \right) + \tfrac{1}{|\partial _x\gamma |} \partial _x(\tfrac{\partial _x \psi }{|\partial _x\gamma |}) \\&= - \langle \partial _s \gamma , \partial _s \psi \rangle \kappa - \partial _s(\langle \partial _s \gamma , \partial _s \psi \rangle \partial _s \gamma ) + \partial _s^2 \psi \end{aligned}$$

and hence by rearranging and the Leibniz rule

$$\begin{aligned} \delta _\psi (\kappa )&= \partial _s^2 \psi - \langle \partial _s \gamma , \partial _s^2 \psi \rangle \partial _s \gamma - \langle \partial _s^2 \gamma , \partial _s \psi \rangle \partial _s \gamma - 2 \langle \partial _s \gamma , \partial _s\psi \rangle \partial _s^2 \gamma \\&= P^\perp _{\partial _s\gamma } (\partial _s^2 \psi ) - 2 \langle \partial _s \gamma , \partial _s \psi \rangle \kappa - \langle \kappa , \partial _s \psi \rangle \partial _s \gamma . \end{aligned}$$

$$\square $$

## Footnotes

1 In fact, the diffeomorphism invariance of the Riemannian curvature tensor naturally yields the Bianchi identities (cf. [17]). Thus, the strongly ellipticity failure of the Ricci tensor is due entirely to this geometric invariance.
